# Abdominal wall desmoid tumors: A case report

**DOI:** 10.3892/ol.2013.1297

**Published:** 2013-04-10

**Authors:** JIN-HUI MA, ZHEN-HAI MA, XUE-FENG DONG, HANG YIN, YONG-FU ZHAO

**Affiliations:** Department of General Surgery, The Second Affiliated Hospital of Dalian Medical University, Dalian, Liaoning 116027, P.R. China

**Keywords:** desmoid tumor, abdominal wall, aggressive fibromatosis

## Abstract

Desmoid tumors (DTs) are rare lesions that do not possess any metastatic potential. However, they have a strong tendency to invade locally and recur. They constitute 3% of all soft tissue tumors and 0.03% of all neoplasms. Abdominal DTs occur sporadically or are associated with certain familial syndromes, such as familial adenomatous polyposis (FAP). The single form of this neoplasm most frequently occurs in females of reproductive age and during pregnancy. A female patient with a DT of the abdominal wall who had no relevant family history was admitted to hospital. The patient, who presented with a painless mass in the left anterolateral abdomen, had no history of trauma, surgery or childbearing. According to the medical history, physical examination and CT report, the patient was diagnosed with DT. Radical resection of the affected abdominal wall musculature was performed, and the defect was replaced with a polypropylene mesh. The histological diagnosis was of DT. The patient remains in good health and complete remission without any other treatment following surgery. DTs exhibit aggressive growth and have a high rate of recurrence. Surgery is the optimal treatment, and subsequent radiotherapy may decrease the local recurrence rate. Further research into their aetiology is required combined with multicentre clinical trials of new treatments in order to improve management of this disease. This case report provides general knowledge of DT, and may be used as a guidance for diagnosis and treatment.

## Introduction

Desmoid tumors (DTs), also known as aggressive fibromatoses, are benign myofibroblastic neoplasms originating from muscular aponeuroses that are also classified as deep fibromatoses (1). They constitute 3% of all soft tissue tumors and 0.03% of all neoplasms (2). Despite their aggressive local infiltration, DTs lack metastatic potential (3). However, the local infiltrations and compressions of surrounding structures demonstrate a high recurrence rate, and in anatomic locations with restricted access to surgical resection, may lead to fatalities (4). DTs usually occur in fertile females and are uncommon during the menopause; during pregnancy an increase in volume occasionally occurs in already existing tumors. This corroborates the estrogen-stimulated tumor growth hypothesis (5). Numerous studies have demonstrated that 37–50% of DTs are initiated in the abdominal area (6). Abdominal DTs occur sporadically or are associated with certain familial syndromes, such as familial adenomatous polyposis (FAP) (7).

This report presents the case of this rare fibromatosis in a 17-year-old female who had no history of trauma, abdominal surgery or childbearing. The appearance of the tumor was analyzed using computed tomography (CT). Informed consent was obtained from the patient prior to the study.

## Case report

A 17-year-old female was admitted to the Department of General Surgery, The Second Affiliated Hospital of Dalian Medical University (Dalian, Liaoning, China) with a painless mass in the left anterolateral abdomen. During a physical examination, the mass was observed to be firm, lacking tenderness and fixed to the abdominal wall. The patient stated that the mass was gradually increasing in size. The patient had no relevant family history and no history of smoking, drinking alcohol or taking any medication. The analyzed blood parameters were within the normal range and the tumor marker results were negative. The patient had no history of trauma, surgery or childbearing.

**Figure 1:**
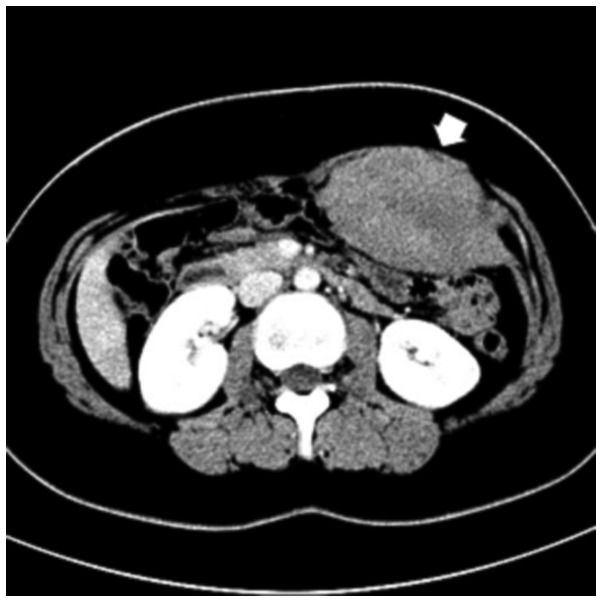
Computed tomography scan with contrast enhancement demonstrating the desmoid tumor originating from the abdominal transversal and internal oblique muscle fascia, with an inhomogeneous formation. Arrow indicates tumor.

Pre-operative CT scans revealed a large mass (9.3×6.1 cm) with unclear borders of attenuation equal to that of muscle. The mass originated from the left rectus abdominis muscle and, following intravenous administration of contrast medium, demonstrated mild enhancement, even in the delayed images (Fig. 1).

**Figure 2:**
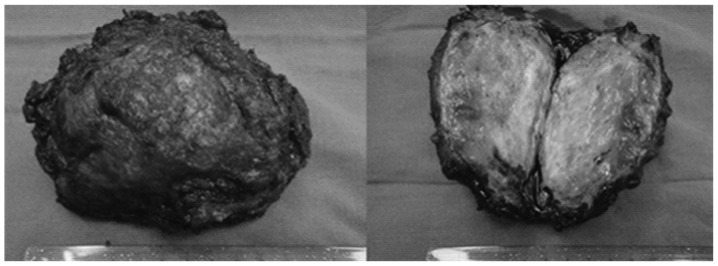
Macroscopic view of the excised rectus desmoid tumor.

**Figure 3:**
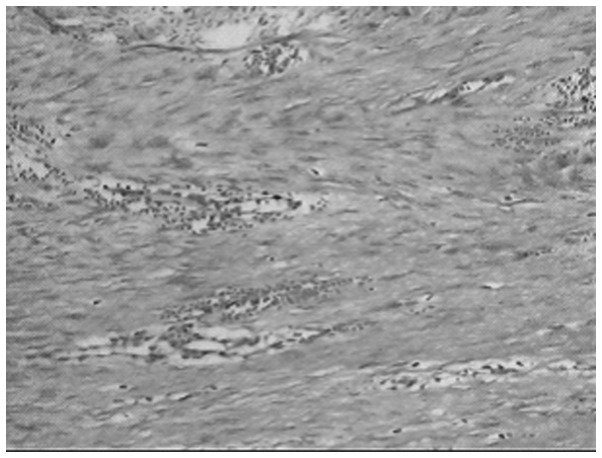
Microscopic view of the excised rectus desmoid tumor. Hematoxylin and eosin staining; magnification, ×100.

Radical resection of the affected abdominal wall musculature was performed down to the peritoneum, and including a peripheral margin of 3 cm of healthy tissue. Following the resection of the DT, the abdominal wall defect was replaced with a Bard Composix EX Mesh (Bard Inc., Cranston, RI, USA). Macroscopically, the tumor had a firm texture. On the cut surface, it was pale and certain areas had the appearance of fish meat. The tumor has no confirmed capsule, and its margin was ill-defined (Fig. 2). The histological diagnosis was of a DT (Fig. 3). The post-operative course was uneventful and the patient was discharged on the ninth post-operative day. After a follow-up of 5 months from the end of surgery, the patient remains in good health and complete remission without any other treatment.

## Discussion

DTs are benign deep fibromatoses that originate from fascia and muscular aponeuroses, with an infiltrating growth pattern (8). DTs are rare tumors with ∼3.7 new cases occurring per one million individuals each year (9). Primarily located abdominally or intra-abdominally (1), DTs have been correlated with the female gender, FAP (10) and occasionally with surgical trauma (11). They have a higher prevalence in females who have experienced pregnancy (12). In the present case, the patient had no history of trauma, surgery or childbearing. Despite their aggressive local infiltration, DTs do not metasta-size to other parts of the body (13–15). Depending on the tumor size, the chosen therapy and the negative resection margins, recurrence is present in ≤45% of cases (16).

Several modern imaging methods, including abdominal ultrasonography, CT and magnetic resonance imaging (MRI), are used for the diagnosis of DTs (17). In ultrasonography, desmoids have a variable echogenicity, with smooth, well-defined margins. In contrast-enhanced CT scans, the tumors are generally characterized by high attenuation (relative to muscle) and have either ill- or well-defined margins. A CT scoring system has been developed, characterizing specimens according to the presence of desmoid precursor lesions (‘mesenteric fibrosis’) and true DTs. This has provided further evidence for a stepwise progression in desmoid development (18). In MRI scans, DTs have a low signal intensity relative to muscle on T1-weighted images, and a variable signal intensity on T2-weighted images (19). MRI scans indicate how the tumors are likely to behave, with a bright signal indicating a high water content, which has been correlated with rapid growth (20). Although there are no specific imaging features to distinguish DTs from other solid masses, the diagnosis of DTs should be considered in patients with an abdominal mass, a history of previous abdominal surgery or injury and where there may be an association with FAP. A definitive diagnosis must be established with a histopathological analysis (21). Characteristically, there is diffuse cell infiltration of the adjacent tissue structures. In addition, the immunohistochemical response for actin may be partially positive, and immunohistochemical muscle cell markers may delimit DTs from fibrosarcoma (22).

The treatment of neoplasms, such as DTs, is guided by their clinical and evolutive characteristics. Radical therapy consists of wide tumor and adjoining tissue resections (23). Surgery has a key role in the management of abdominal DTs; the resection of abdominal wall (superficial) DTs is simple and may be performed safely when the lesion is growing and possesses clear margins (24,25). Incomplete resection is correlated with high recurrence rates. Abdominal wall reconstruction may be achieved by direct repair (with sutures), or by using synthetic materials (meshes) or myocutaneous flaps when the defect is extensive (26–28). In the present case, the peritoneal defect was replaced with a Bard Composix mesh. Prosthetic materials are more susceptible to bacterial infection and other complications (29,30), although newly developed materials have exhibited encouraging experimental results (31). Radiation therapy has been used predominantly for the treatment of extra-abdominal DTs, and has resulted in an improvement in the local control of DTs by reducing local recurrence rates (32). External beam irradiation or brachytherapy may be used alone, predominantly in patients with inoperable lesions (33), although they are correlated with high failure rates (34). They may also be used either prior to surgery, or as adjuvant therapies following incomplete (non-radical) surgical resection.

In conclusion, the optimal treatment for DTs remains unclear. Surgery is the primary treatment option, although it carries a risk of local recurrence. A radical resection with clear margins remains the principal determinant of outcome. The repair of abdominal wall defects may be achieved with prosthetic mesh reconstruction, which leads to good functional results. Non-surgical treatments result in diverse and unpredictable outcomes, but are considered as an option for adjuvant therapy in patients with unresectable lesions.
